# Psychobiology of Attachment and Trauma—Some General Remarks From a Clinical Perspective

**DOI:** 10.3389/fpsyt.2019.00914

**Published:** 2019-12-12

**Authors:** Theresa Lahousen, Human Friedrich Unterrainer, Hans-Peter Kapfhammer

**Affiliations:** ^1^University Clinic for Psychiatry and Psychotherapeutic Medicine, Medical University Graz, Graz, Austria; ^2^Center for Integrative Addiction Research (CIAR), Grüner Kreis Society, Vienna, Austria; ^3^Institute of Religious Studies, University of Vienna, Vienna, Austria

**Keywords:** attachment, secure-insecure, disoriented-disorganized, oxytocin, empathy, mentalization, trauma, neurobiology

## Abstract

Attachment refers to a psychobiological principle that is deeply rooted in evolutionary development; it is thought to contribute a major advantage in the survival of the social group. Within individual development it indicates a primary motivational system that guides the initial transactions between mother and baby and furthermore mediates affective attunement and regulation. Psychosocial learning, in close interaction with genetics and epigenetics, also develops a decisive foundation for further brain development of the infant. Finally, the attachment pattern established forms an enduring, relational context for later affective, cognitive, and social development of the child. As an unconsciously active matrix for future personal relationships it has a particular impact on the comprehensive psychological functions of empathy and mentalization. Early adverse and traumatic experiences or major emotional neglect may lead to different levels of security versus insecurity or disorientation-disorganization of the attachment pattern that corresponds to characteristic features of neurobiological regulation.

## Introduction

Early representatives of psychoanalysis argued that the roots of human social motivation are primarily physical and sensory (hunger, sexuality) and that satisfaction and/or frustration of these needs lead to the infant’s initial approach to the mother. In this theoretical view, attachment refers to a “secondary motivational system.” The British pediatrician, child psychiatrist, and psychoanalyst John Bowlby (1907–1990) strongly opposed this theoretical approach. Based on numerous empirical observations he developed a different theory: the infant’s hunger for its mother’s love and presence is as great as its hunger for food. Attachment is therefore a “primary motivational system” with its own workings. In a remarkable study conducted for the World Health Organization (WHO), Bowlby (1)provided substantial arguments supporting his view. He emphasized the importance of the link between the quality of maternal care and the child’s future mental health. Leading psychoanalytic representatives initially fiercely dismissed Bowlby’s position, in spite of the fact that Rene Spitz (2)had already made similar empirical observations with orphaned children some years earlier. Although carers in orphanages adequately met children’s basic nutritional and hygienic needs, they failed to deliver reliable emotional support. The development of these children demonstrated that the care they received belied a disconcerting, psychosomatic failure to thrive, in addition to a high mortality rate. Early ethological research supported Bowlby’s theory. Konrad Lorenz (1903–1989) had discovered that ducklings develop a strong bond towards their mother, even if she did not feed them (3). Harry Harlow’s (1905–1981) thrilling experiments on rhesus monkeys proved that the need to satisfy hunger is important, but the need for social contact is greater. Any prolonged separation from the mother, as well as an isolated upbringing, led to massive impairments of social behavior. Harlow also suggested that not only maternal bonds but also other social interactions e.g. playing with peers of the same age, were apparently crucial as regards further socioemotional development (4).

Bowlby (5) verified his attachment theory with some pioneering publications: the bond between infant and primary caregiver, usually the mother, refers to a deeply rooted evolutionary system of interaction, which increases the infant’s chances of survival. Along with the infant’s emotional and cognitive development and the care of its mother, a strong emotional connection is gradually developed under favorable conditions. He distinguished four stages of early development of attachment: 1) *Preattachment* (birth to 6 weeks): the infant is in close contact to its mother as well as other family members. It indicates a strong need for closeness. 2) *Early arising attachment* (6 weeks to 6–8 months): the infant reacts differently towards people it knows than those it does not; basic trust is developing. 3) *Clear-cut attachment* (6–8 months to 18–24 months): the infant shows separation anxiety and a clear attachment to its caregiver. 4) *Differentiation and integration of attachment* (18 months to >2 years): the toddler creates a reciprocal relationship with its caregiver. It actively participates in interactions, asks questions, and negotiates. Furthermore, Bowlby (5) suggested that early attachment experience creates internal working models as “life-long templates.” These templates create an affective as well as cognitive matrix for future relationship patterns. Based on his theoretically sound and clinically tested results Bowlby succeeded in establishing his own tradition of attachment theory.

Mary Ainsworth (1913–1999), a co-worker of Bowlby, initiated an early empirical approach. To evaluate the quality of attachment formed by children between the ages of 1–2 years, Ainsworth created a valuable psychological system of assessment. Based on her method there are three key criteria for creating attachment bonds: 1) proximity seeking to caregiver, 2) effect of a “secure base,” and 3) protest when separated from the caregiver. Her paradigm of “*separation and reunion*” refers to a characteristic research situation: a mother and her child enter a room, which offers a large number of toys. In the next scene a stranger, usually a research assistant, joins the pair. The child starts exploring its environment; it picks up toys and engages in play. Next, the child initiates contact to the stranger. The mother, still present, is sitting close by while reading the paper but is still open to contact if initiated by the child. After a while, the mother leaves the room. The behavioral and emotional reactions of the child to the separation of its mother as well as the reunion are the focus of the experiment. According to attachment theory, children between the ages of 12–20 months should have developed a secure and unique attachment to their mothers as a normative developmental milestone. Thus, even a brief separation in this crucial developmental stage can cause tremendous emotional suffering. Ainsworth’s, (6) research has shown that:

- Children with a “*secure attachment*” protest when left alone. They show major distress, often cry, disrupt their play, and feel discomfort in the presence of a stranger. When the mother returns the child seems joyful, seeks proximity, calms quickly, and returns to play.
- Children with an “*anxious-avoidant* attachment” do not protest when the mother leaves the room. They continue to engage in play and initiate familiar contact with the stranger. At first glance, these children do not seem to feel uncomfortable or anxious. They do not react or notice the return of the mother and turn away if the mother tries to approach the child. This seemingly mature behavior, however, is in fact accompanied by significant internal stress. This behavioral strategy is supposed to turn the child’s attention away from the situation of separation to avoid any correlating emotional pain.
- Children with an “*anxious-ambivalent attachment*” react with tremendous emotional distress when being left alone, similarly to securely attached children; they seek strong physical contact when the mother returns. Their behavior toward the mother, however, is ambivalent and alternates between clingy and avoidant. Their disproportionate attachment behavior appears to follow a strong urge to control the uncertain attachment with the mother.
- Children with a “*disoriented-disorganized attachment*” lack any consistent pattern in response to the separation and return of the mother. Children displaying this type of attachment are confused by separation, throw tantrums, and are inconsolable. When the mother returns, the child seems to, simultaneously, seek proximity and avoid the parent. Numerous idiosyncratic behavioral mechanisms such as freezing, stilling, and other psychomotor stereotypes are apparent.

These various attachment styles reflect the history between a mother and her child during early childhood. They are highly predictive of future relationships. Research suggests that the majority of children, who grow up under overall positive interpersonal and social circumstances, form a stable attachment security that endures throughout their life (7). In general, probability for attachment security increases if parents consistently meet their children’s needs in a sensitive manner. Attachment security is a crucial factor for the development of children’s emotional, cognitive, and interpersonal competence. On the other hand, exposure to trauma in early childhood significantly interferes with the ability to form secure attachments. Despite experiencing trauma such as neglect and abusive behavior, however, all children continue seeking proximity and develop distinct attachment patterns (7).

The present work deals with psychobiological aspects of early interactions between toddler and the primary parental figure, usually the mother, which, in a processual manner, lead to the establishment of a distinct attachment pattern with fundamental implications also on the further affective, cognitive, and social development of the child in later stages of life. It first places “bonding” in a more general evolutionary context. Thus, the developmental principle “attachment” can already be discovered in primitive forms of a cellular differentiating organic life. Central to this is the oxytocin system, which undergoes numerous transformations in the course of phylogenesis. It achieves crucial adaptive progress when it is eventually linked to a reward system as the brain structures become phylogenetic. The importance of oxytocin is specifically linked to the emergence of important social contacts and attachments in general, for the attachment system unfolding in early baby-mother interactions. Empirical studies emphasize highly complex interactions on a psychological as well as a neurobiological level. Genetic programs of the child, ongoing experiences in the mother-child dyad and the familial and social environment, as well as epigenetic processes triggered thereby must be coordinated with each other in fine orchestration, so that ultimately a secure attachment pattern can emerge. The closely related concepts of cognitive and affective empathy on the one hand and of mentalization on the other are outlined in the context of the fundamental bonding patterns in a subsequent section. This is followed by a summary of empirically established insights into neuronal, structural, and functional relationships, which are currently being discussed as typical of safe versus unsafe attachment patterns.

Furthermore, this work primarily aims to provide a prototypical description of interactive and intrapsychic processes underlying distinct attachment patterns. It takes up findings from different developmental psychological and psychopathological examination contexts. From a clinical perspective, they are considered to be of exemplary use for understanding frequent deficits in empathy and mentalization in adult patients with different impaired attachment patterns. This form of presentation also involves a number of theoretical and methodological problems that need to be discussed. The authors are self-consciously aware that a clinical focus on prototypical principles in the psychobiology of attachment represents a strong shortening and simplification of those highly complex relationships governed by numerous mediator and moderator variables, as theoretically conceptualized in the transactional models of modern developmental psychological methodology (see 8).

### Attachment From an Evolutionary Perspective

The great Russian-American geneticist, zoologist, and evolutionary biologist Theodosius Dobzhansky (1900–1975) once said, “nothing in biology makes sense except in the light of evolution” (9, p.125). Therefore, the quest for the evolutionary root of attachment and its connection in well-established adaptive patterns and mechanisms of its implementation arises. In an overall evolutionary sense, four principles of behavior can be distinguished: 1. maintenance of homeostasis, 2. consumption and acquisition of energy, 3. prevention of harm, and 4. reproduction.

This evolutionary program implies underlying primary motivational systems, which connect emotional responses with distinctive tendencies of action that are correlated with distinct affective qualities of experience. These primary motivational systems are rooted in the phylogenetic organization of the brain. This form of organization considers exclusive internal appetencies as well as environmental cues signaling either danger or reward (10). A genetically rooted preparedness to rapid action, however based on low information processing, is gradually replaced by more complex decision making, planning and behavioral execution. These developmental stages advance not only in accordance with endogenic growth, but continuously refer to manifold social learning processes within a primary relational context. Above all, they orchestrate the care of the parents for their children and provide the base for a life-long attachment, thus guaranteeing affective communication as well as social relationships (11).

Tomkins (12, 13) psychophysiological affect theory suggests a fundamental view of the development of basic emotions in early childhood. According to Tomkins, infants possess a highly differentiated set of basic emotions by the time they are born. Distinctive motor and visceral reactive patterns are connected to specific primary affects. Through subtle facial expressions, a set of eight primary affects may be distinguished. Low and high intensity labels characterize these affects [positive: interest/excitement, enjoyment/joy, surprise/startle; negative: distress/anguish, fear/terror, shame/humiliation, disgust/dissmell, and anger/rage]. Affective neuroscience suggests that primary affects can be associated with distinctive neuronal regulatory circuits. They may be identified as phylogenetically acquired adaptive structures that are widely shared among primates; in preliminary stages they reach back even further (14).

The main affective structures are directed to specific adaptive goals. Ontogenetic value is underlined through focusing attention onto significant stimuli, avoiding automatic reactions to harmful stimuli, allowing the recognition of disappointing objects, preventing overwhelming discrepancy in information processing and, most importantly, promoting parent-child proximity to ensure survival. The main affective structures form primary motivational systems when typical perceptions cause characteristic neuronal action potentials according to the gradient of the stimulus change, its absolute intensity and duration. Their biological function aims to frame stimulation in a general and abstract manner and furthermore provides it with a distinct affective, analogue quality of experience. Thus, emotions translate and amplify stimuli from various sources such as biological drives, pain signals and external perceptions along with thoughts, ideas and other affects. Defined affects show differences in facial expressions, biosocial information transmitted, as well as differences in tolerance and coping. During individual development, these affective components may undergo independent developmental trajectories (15). The system of attachment assumes a superior role during early affective development. According to the phylogenetic structural program, individual regulatory principles such as affective arousal, emotional understanding, and emotional behavior are acquired which, subsequently, are transformed into a subjective competence through continuous interactions with the primary caregivers (i.e. the parents), mainly the mother (16, 17).

### Hypothetical Advantages of Attachment Respectively of Eusociality

A hypothesis of anthropological evolution implies (18): During evolutionary progression of the hominid to the homo sapiens, larger social groups have acquired vital developmental advantages. To facilitate and process complex social information, a larger brain volume was necessary. More complex brains required longer periods for an individual to develop, most notably in the postnatal period. Hence, a prolonged period of infant dependency resulted. Longer periods of dependency required additional parental attention as well as greater supervision, specialized childcare and supportive social structures. Growth of social groups facilitated the development of language; basic vocalization and gestures were insufficient for social communication. Thus, development of language subsequently set in. In turn, complex social structures initiated the development of more complex structures of communication. Ultimately, spoken and written language developed.

### Evolutionary Development According to “Attachment Theory” and “Eusociality”

Numerous molecular studies allow a more precise understanding of the evolutionary development of attachment; for example, it is accepted that the oxytocin system plays a major role in a pro-social system. In its precursor stages, this system is traceable down to metazoa, which in contrast to protozoa already feature signs of differentiated and specialized cells, being an early multicellular organism. Over the course of millions of years, the oxytocin system has undergone numerous evolutionary transformations through the various stages of vertebrates to mammals and hominids and finally to homo sapiens. Various successive developmental steps of precursor cells, precursor peptides, as well as precursor receptors of oxytocin characterize this evolutionary progression (19).

During the course of evolution, oxytocin achieved a crucial developmental milestone by linking the attachment system to the reward system. Thus, attachment and love as well as care and its underlying parental bonding process towards the infant started to become rewarding. Interestingly, differential sensory information in various species has regulated both the attachment and reward systems during this evolutionary process. Thus, acoustic signals distinctively activate the attachment system in birds while olfactory stimuli that of rodents. Humans’, as well as other mammals’, attachment system is primarily activated through visual stimuli (19).

### Neuroanatomical Pathways Underlying the Complex Effects of Oxytocin

While gazing at an infant’s face, hypothalamic parvocellular and magnocellular neurons as well as nuclei supraoptici release oxytocin and vasopressin in the mother’s brain. Oxytocin and vasopressin enter different brain areas, such as the anterior pituitary lobe, amygdala and brainstem (20). Simultaneously, through a complex interaction between various neurotransmitter systems such as opidoid and dopaminergic, the reward system is activated (ncl. accumbens; ventral tegmental area; ventral striatum) (21, 22).

Oxytocin and vasopressin’s genetic information are located on adjoining gene-sequences and differ from each other by only two amino acids. Overall effects of oxytocin and vasopressin are manifold. They facilitate both affiliative-nurturing behavior and aggressive-defensive behavior towards external threats against attachment. Oxytocin may also lead to anxiety related reactions, in addition to regulating homoestatic energy levels and modulate perception of pain. A coordinated orchestration of HPA axis, opioids, dopamine, serotonin, norepinephrine and glucocorticoids mediate these complex functions (23).

### Overall Effect of Oxytocin for the Development of Close Social Bonding

Oxytocin directs overall social interaction behavior on a neurobiological level. Dopamine initially released during a social interaction in the ventral tegmental area determines the degree of attractiveness of this contact (“*wanting*”). Activation of DA_2_ receptors in the nucleus accumbens leads to the release of opioids, which in the ventral striatum and prefrontal cortex (PFC) cause this contact to be positive and rewarding (“*liking*”). On the other hand, up-regulation of DA_1_ receptors seems to be crucial for the maintenance of such a contact (social bonding). Oxytocin, in combination with vasopressin, determines selective bonding, which may also include increased aggression towards rival sexual partners. Through attachment to a partner, an overall social stress buffer and unique partner protection will result. At the same time, strong negative emotional stress occurs when the partners temporarily or completely separate. Separation pain, longing, and sadness may occur. At the neurobiological level, a unique organization of oxytocin receptor, arginine-vasopressin-receptor_1a_, and DA_1_- and DA_2_- receptors has been evolutionarily established in a coherent manner to this complex social behavioral regulation (22).

### Psychobiology of Early Attachment

From the 2nd/3rd trimester onwards, mother and embryo are increasingly attuning to each other under the dominant influence of oxytocin. A major fall of progesterone right before birth continuously releases oxytocin and thereby triggers labor activity. Simultaneously, oxytocin relieves pain during labor. Following delivery, the new-born’s suckling on the maternal breast promotes secretion of both prolactin and oxytocin. Breastfeeding provides a harmonious, highly satisfying and stress-reducing communication between baby and mother. Oxytocin significantly contributes to the development of a sense of security and social connectivity; it promotes the selective attention of the mother; causes contact, closeness, warmth, and love between mother and child. Oxytocin is associated with a passive, vasopressin with an active coping pattern. Oxytocin also shows significant anti-inflammatory and antioxidant effects. Furthermore, oxytocin has been shown to have an impact on additional steps of the baby’s further brain development: Triggered GABAergic neurotransmission promotes synchronization in the cortex and hippocampus and thus co-organizes social learning. Empirical evidence underlines the fact that the development of the infantile brain unfolds under the critical influence of social experiences. There is close interaction with genetic programs and epigenetic processes initiated by ongoing relational and social experiences (24–26)

Meaney, (27) numerous animal studies have given a fascinating insight into the high complexity of this early mother-to-child dyad and its consequences for the development of the brain: genes organize the brain and trigger sensitive phases. Current social experiences orchestrate genetic transcription in a continuous adaptive organization of neural systems. However, it is the specific quality of these early social relationship experiences that activate key epigenetic mechanisms and thereby very specifically modify genetic expression, particularly through DNA methylation or changes in the chromatin structure. Growth and plasticity of the neural organization of the child’s brain is not the only developmental step controlled by this process. It additionally programs long-term future stress responsiveness, particularly *via* modulation of HPA activity. It should be noted that through a direct transmission from the mother even the daughter’s future maternal behavior is being already pre-primed by impact on growth and activation of her medial optic area and the regulation of oxytocin and estrogen receptors.

These and several other neuroscientific research findings demonstrate that early mother-baby attachment is the key interpersonal and social matrix for the child’s brain development. It thus establishes the child’s future potential for mental development and physical health on the one hand and mediates a variety of mental and somatic disease risks on the other. Winnicott (28, 29) so inspiringly introduced the psychodynamic concepts of “primary maternal care,” “good enough mothering,” “mirroring,” “holding environment,” and “ability to be alone in the presence of the other” decades ago, into psychoanalytic developmental psychology. His terms today may find their immediate neurobiological correlation in the development of relational and social cerebral maturation (30–32).

### Prerequisites for a Successful Attunement and Affect Regulation in the Attachment System

From an evolutionary perspective, it is imperative to understand what other members of the same social group think and feel, to be able to communicate and share affective states with them, make predictions about other people’s intentions and understand possible motives for their actions, and in general to show prosocial behavior. The global term of “empathy” refers to several abilities that map overlapping but differently structured functions. These closely relate to the various forms of affective perception, experiencing, understanding and communicating. They provide the basis for socio-affective action in a hierarchical organization:

- Thus, *affective contagion* describes a tendency to absorb the emotional state of another person without having to understand the reason for the emotional experience itself.
- *Mimicry* means to synchronize one’s own facial expressions, vocalizations, gestures, postures and movements with those of another person, but without feeling the same emotion of the other. Affective contagion and mimicry do not require a clear separation between self and object.
- *Imitation* refers to a purposeful act observed on another person. However, it does not necessarily correspond to the actual affective state of this imitated person.
- *Emotional empathy* requires a clear differentiation between self and object, and imbeds to feel how the other feels.
- *Cognitive empathy* involves knowing what the other person thinks, feels, intends and why. Emotional and cognitive empathy critically imply a reflective self-awareness.
- *Sympathy* means positive feelings for another and strives for the other to get better. It is a prosocial motivation based on empathy. It involves a more cognitively determined mental process, which succeeds through taking over the other person’s perspective and leads to interpersonally shared feelings and goals.
- *Compassion* involves empathic concern for the other and motivates one to care and console. It does not necessarily presuppose a common, identical feeling with the other.
- *Empathic concern* is an emotional and motivational condition that seeks to help and to contribute to the well-being of others.

Furthermore, all of these aspects of perception, understanding, communication and the regulation of affective life in relationships are anchored in different neural systems of the brain (33).

*Affective empathy* concerns phylogenetically older parts of the brain and is initially based on an affective transference, which essentially comes about through the activation of mirror neurons. Mirror neurons were first detected in monkeys in the supplementary motor area. They specifically fired when monkeys observed motor actions of conspecifics in the environment. This neural mechanism suggests an evolutionary heritage representing an interactive understanding of action, at least in the primate range (34). In humans, this system is much more complex (35). It allows the representation of another person’s emotional state that may be shared by an in-body simulation (36). In a neural network the inferior part of the parietal lobule, posterior superior temporal sulcus, premotor cortex, and especially the anterior part of the insula and anterior and middle sections of the cingulate cortex (ACC, MCC) functionally interconnect for this performance. The latter two structures occupy a special position. Insular cortex is responsible for the representation and integration of internal visceral and emotional states (37). Insula maps global emotional states, includes uncertainty information and risk preferences and conveys self-awareness. It has important connections to the prefrontal and cingulate cortex as well as to the temporal lobe, limbic system, thalamus, basal ganglia and the brainstem. ACC represents insular information on a higher cognitive level and coordinates reality-oriented decisions and actions. In particular, posterior parts of the ACC and anterior parts of the middle cingulate cortex seem to be crucial for the apprehension of another person’s pain experience (17).

Another distinct neuronal network, in turn, mediates *cognitive empathy*. It functionally joins the ventromedial prefrontal cortex, temporal pole, posterior cingulate cortex, precuneus and temporoparietal junction. This system allows for complex cognitive operations such as assessing what another person thinks, feels and intends in a defined situation, and what the reasons might be (38).

Closely related to these different, basal and also higher structured levels of the empathy-concept is the concept of *mentalization*. In recent decades mentalization has been fruitfully implemented in psychoanalytical and socio-cognitive developmental psychology, in research involving trauma and posttraumatic disorders and furthermore has led to highly promising clinical-therapeutic approaches for various psychopathologies by the research group around Peter Fonagy. Again, the mentalization concept also includes various dimensions. These dimensions may be characterized by several polarities: by an automatic experience mode, very likely evolutionarily anchored versus a controlled one, strongly based in social and interactive learning, by a predominantly external versus predominantly internal orientation, by a focus on the self versus one on significant others, by a primarily affective versus a primarily cognitive mentalization. These dimensions are not intended to be understood separately. They are hierarchically coordinated against the background of relatively undisturbed developmental conditions and are used flexibly depending on the requirements in normal, everyday situations. However, these dimensions may functionally dissociate under special conditions. These dimensions can also be assigned to differential neuronal functional systems (39). Not surprisingly, the neural systems of mentalization essentially overlap with those underpinning the concept of empathy. Even if current knowledge is based on aggregated data from numerous empirical studies, their provisional, hypothetical character should be considered.

With these conceptual prerequisites outlined, some fundamental processes of the attachment system can be considered in greater detail, which is established between baby and mother in a different distribution of tasks. It refers to a complex system of interactive affect attunement and regulation, which in its earliest stage may rely on evolutionary mechanisms:

- Primary affects are promptly and automatically available to the infant to deal with “*common situations*” (see above). However, this adaptive function of the primary affects creates the problem of an “individual reality” that does not necessarily always coincide with the circumstances of a particular situation, i.e., the system of primary affects must be relativized by a corrective system of coherent perceptions and cognitions in further development.
- Primary affects are always discrete messages to the mother. The infant’s differentiated affective expressions also have a differentiated quality of subjective experience at the level of physical sensation. Despite the attribution of a differentiated experience of meaning, this does not yet imply a self-reflexive awareness. Subjective experience of meaning in the sense of meaningful feelings always requires the prior acquisition of a self-concept that arises in the course of the second year of life at the earliest. Self-reflexive experience of feelings is only possible at the level of a symbolically represented self. The development of the child’s affect system initially receives its full meaning only in a systematic relation to the transactional system of the mother-child dyad.

Fonagy and co-workers, (40) consider “*mentalized affectivity*” to be the most mature form of affect regulation, favorably acquired through several years of development into adolescence and beyond. It describes the highly differentiated ability to attribute one’s own feelings to subjective meanings and to be able to use them constructively in interpersonal relationships. However, their developmental origins lie in the mother’s early reflection of the child’s affective state. It is therefore rooted in the attachment process itself. The infant’s automatic affect expressions and the maternal affective responses *via*her facial expression and voice are linked by a constitutionally anchored contingency detection mechanism. On the one hand the infant succeeds in exerting control over the maternal mirroring behavior and, on the other hand, in experiencing feelings of well-being in its own emotional state. At the same time, affect mirroring also serves as the basis for the development of a representational framework in which the infant’s affects can be recognized as subjective manifestations of self-organization. In order to allow the infant to maintain a regulated state in the first few months of life, the most exact recognition of affection with the highest degree of contingency by the mother is necessary. This pattern of affective mirroring changes after the third month. Although the mother’s reaction must be congruent with the infant’s affect expressions and should capture it empathetically, her facial expressions and vocalizations should mark the baby’s expression of affect by a certain exaggeration thereby reflecting it back. The baby internalizes the empathetic behavior of the mother towards his/her state of affect, thereby gaining a representation of a second order of his/her own affectivity. Here, the empathetic maternal face is the bearer of meaning, but the emotional arousal of the child becomes significant. The maternal affective response alters the infantile emotion insofar as it modifies the primary experience and modulates it in its potentially disorganizing intensity, thus organizing the child’s self-state.

### Characteristics of Secure Attachment

In these early reciprocal affective processes of the “*self with the other*,” the interaction of affects is directed at three essential goals (41): 1. a reliable state transformation of the baby from an aversive state (e.g., hunger) to a comforting, satisfied state (e.g., satiety, consolation), 2. a playful interactive behavioral sequence of baby and mother in mutual relaxation, 3. a deep affective attunement, a shared, positively experienced emotional state.

Trust, reciprocity, intimacy, and love are higher structured psychological qualities of affective experience in such a primary relational context. In a neurobiological perspective, these successful affective exchanges are not only the basis of attachment; they are also motivationally coupled with the reward system (42). The main trajectory of this neural network extends from the limbic structure of the ventral tegmental area to the nucleus accumbens in the ventral striatum and is closely related to the amygdala on the one hand and the prefrontal cortex on the other. Thus, the evolutionarily highest system of stress regulation has been interactively practiced (31):

- In the case of imminent danger, the most recent part in evolutionary terms in a hierarchical sequence is activated, e.g. the ventral vagus complex, the nucleus ambiguus and the motor cranial nerves; this initiates a social orientation reaction, a turn towards a familiar face, a contact search with vocalization that allows for verbal communication.
- When this response of the social contact system does not lead to a signal of security, sympathetic reaction patterns of fight and flight are mobilized.
- In traumatic situations, which emphasize hopelessness in addition to states of helplessness, the oldest phylogenetical neural response system is activated, namely the dorsal vagus complex, which blocks essential motor-aggressive defensive movements, and leads to immobilization, passive avoidance, and freezing in a dissociative state.

In further behavioral organizations, therefore, the attachment system is associated with both the reward system and the fear-anxiety system, as well as higher instances of behavioral selection and modulation of response intensity. The experiences accumulated during the early affective exchange processes between baby and mother serve as enduring affective-cognitive models of attachment especially regulated by the right-hemisphere orbitofrontal cortex. As an unconscious neuronal relational blueprint, they will essentially frame future interpersonal contacts and partnerships. In acute states of affective union between mother and baby, but also of intimacy and falling in love with partners, the close connection of the attachment system to the reward system becomes apparent. As already described, these intimate affective exchange processes are promoted on the one hand by the central hormones oxytocin and vasopressin, and on the other hand mediated by the neurotransmitters dopamine, serotonin, and opioids. However, as a downside of these highly rewarding affective interactions, higher cortical areas are temporarily deactivated (“*love makes blind*”) (43, 44). Affective empathy is pioneered above all in these acute states of “*being one*,” cognitive empathy requires “*calmer moments*.” These particular emotional moments also facilitate the later development of basic self-reflective skills that are predominantly mediated by prefrontal cortical structures (39).

The child gradually acquires these higher-structured cognitive empathy and mentalization skills during prolonged sociocognitive and affective development in the context of a secure attachment (40):

- Before the child learns that its inner states actually represent the external reality symbolically, it experiences the inner and the outside world as psychologically equivalent. What it experiences in its fantasy, it equally expects in its outward orientation and vice versa (*mode of psychic equivalence*). This can be intimidating, even frightening for a while, when feelings and ideas are considered as external objects. The repeated experience of a contingent, congruent, and well-marked mirroring of the child’s emotions by the parents contributes to the gradual realization that one’s own affects do not necessarily pass on to the outside world, but can be disconnected from physical reality and have a subjective dimension.
- This experience situation especially manifests in free play. As the child plays, it can manipulate and transform objects of reality according to its inner needs, without any concrete impact on external reality in this transitional space (*as-if mode*). When parents accompany the child in play, non-intrusively directing its attention and encouraging constructive solutions in a commonly shared focus, they securely anchor the child’s perceptions and feelings with the outside world.
- The child gradually learns to perceive himself as an *intentional*agent. His concept of self initially confines itself to the physical-somatic sphere and then gradually expands to the social sphere of interaction. In the distinction between means and ends, action and result, it acquires a teleological standpoint. This allows effectively controlling instrumental behavior in many everyday situations. In complex interpersonal relationships, conflicts and emotional tensions, however, the narrow limits of this position become apparent.
- The high-structured ability to conceive of oneself as an actor in motivic concepts of inner states may only be acquired in a socio-cognitive development that continues for several years. A mature stage of *affective and cognitive mentalization* is based on the firm realization that other significant partners of interaction are determined by independent subjective motivations in their own actions, and that they can also be influenced by subjectively motivated, intentional actions by oneself.

### Characteristics of Insecure Attachment

Not all early interactions between a mother and her infant are inherently, consistently ideal, just as later relationship experiences are not always conflict-free. A hallmark of a secure attachment is that the mother-child dyad succeeds in regaining much of the above-described satisfactory affective transformation as well as efficiently overcoming painful disruptions in the relationship. An insecure attachment, on the other hand, is the result of mostly unsuccessful early affective coordination processes. This may be the result of an emotionally unstable and probably insecurely attached mother, or related to an inherently difficult temperament of the child**.**

A secure attachment system unfolds differently compared to an insecure attachment system. A striking clue may be observed by comparing securely versus insecurely attached mothers with each other in a behavioral observation environment as they look at images of their own baby versus those of an unknown infant in different states of affect (happy versus distress). The most striking difference is underlined: Insecurely attached mothers seeing their baby in a well-balanced state results in significantly lower activation of their reward system and associated neural relationship representations (fMRI: activation of the right ventral striatum, activation of the ventromedial PFC). For securely attached mothers, the reward system is activated significantly (fMRI: activation of the right ventral striatum) even when they look at a picture of their baby in a crying, unhappy state. Insecurely attached mothers, on the other hand, are largely detached from the reward system; instead, there is a prominent activation of the right dorsolateral PFC in this condition, which may be interpreted as an intensified effort to deal with this irritating situation. Securely attached mothers manage this challenging task of comforting their unhappy baby intuitively, with the certainty of being able to create a harmonious condition for it. Obviously, the difference in maternal performance depends on the level of oxytocin measured (45).

Oxytocin regulates the general gaze behavior of the mother towards her child, intentionally looking at the baby’s face as well as avoiding it. Securely-attached mothers have a high situational oxytocin level and maintain intensive eye contact with their baby for a longer duration than insecurely-attached mothers with lower oxytocin concentrations, who additionally have a significantly higher rate of eye contact avoidance and vision loss (46). The amygdala is significantly involved in the recognition and affective evaluation of emotional facial expressions. Both the negative and the positive emotional facial expressions activate it. In a group of first mothers, the sight of one’s own baby generally activated the amygdala more than that of an unknown baby. The activation is also generally more intense at the sight of the baby in a happy state than in an unhappy state (47). It seems relevant that insecure mothers show markedly reduced amgydala activation (blunted response) when their baby is in a state of subjective distress, which may be interpreted as evidence of emotional detachment (48).

The neurobiological starting point of insecure attachment processes in the early mother-child dyad thus differs significantly from that in a secure attachment situation. Several aspects may be highlighted schematically (49): Numerous interactions in insecure attachment systems are mentalized to a lesser extent. Overall, both partners consider the interactions as less rewarding. Oxytocin-mediated coupling of the attachment and reward system is significantly impaired while also providing less protection against stressful situations. A sensitization of the HPA axis through epigenetic mechanisms lays the foundation for a long-term increased susceptibility to stress. A reduced production of BDNF in turn means less favorable conditions for the neuroplasticity needed for a higher structuring of affective and cognitive functions in further development.

The intrapsychic inheritance of such insecure attachments is manifold (40):

- Non-contingent affect reflections by the mother may cause wide areas of the child’s affectivity to remain undifferentiated and not clearly represented in subjective self-experience. The mother often perceives even calm, positive experiences with less joy and attunement. To a lesser extent, recurrent interactions lead to that characteristic happy framing of the formative scenes of “*togetherness*,” which usually build the foundation for trust, self-esteem, and intimacy. The temporary loss of a balanced psychophysical state of the child, in which it begins to cry or be sad, often presents the mother with conflicting challenges that endanger a secure transformation of the child’s stressful state. Above all, non-containing and incongruously marking reactions of the mother, leading for example to inappropriately excitatory, anxious-worried or dismissive affect reflections on the mother’s side, prevent the development of stable affect representations of the second order on the child’s side. They undermine the secure creation of a boundary between self and object representations in the child’s inner world. Such affective communication, which essentially involves the mother’s actual, unconcerned affective experience, may act like an “*alien self part*” in the maturing self-organization of the child. It can form an affective behavioral disposition, which, especially under stress, presses for externalization in a concrete relationship situation in order to re-establish a precarious self-coherence for oneself. More cognitively overshadowed schemata of unsecure, worthless, ashamed, guilty self versus schemata of unreliable, dangerous, confusing, rejecting objects may also be a consequence.
- An integration of the early modes of the perception of reality of “*psychic equivalence*” and “*as if*” usually fails in this context of insecure attachment. Play and fantasy activity in the transitional space may unfold only poorly. If the outside world is experienced as analogous to the inner world and vice versa, then there is a high degree of vulnerability to suddenly intense and, in principle, traumatogenic affects. By contrast, an increasingly defensive retreat to an “*as if*” mode means a predominantly dissociative attitude of protection that may severely inhibit a constructive engagement with social reality.
- An *intentional* stage of self-development in the sense of a mature affective and cognitive mentalization is usually missed as well. Instead, there prevails a *teleological* point of view, especially in close relationships. Reaching a dominant goal often seems to justify any means. If the subjective goal is, for example, to deal with an overwhelming fear or a basic shame, above all aggressive, self-directed and object-directed affects and actions produce some form of self-coherence. However, this implicitly destructive dimension of one’s own actions cannot be critically reflected on the self and negative effects on the partner involved cannot be adequately assessed.
- In an insecure attachment, primary caregivers have proven to be unreliable, poorly predictable, and hardly positive for the child. This child subsequently shows a strong sensitivity and hypervigilance towards potential threats in the social environment. Situations of inner tension and interpersonal uncertainty always activate the established attachment system and the associated system of mentalization functions. However, not all functions of mentalization are available in every social situation and do not always allow effective strategies for affective regulation and cognitive orientation.

A controlled affective and cognitive mentalization is no longer possible especially with increasing stress levels. From a neurobiological perspective, mature mentalization achievements can only succeed up to states of a moderately elevated arousal. D_1_-dopaminergic, α_2_-adrenergic, and serotonergic neurotransmission usually provides an approach behavior. A coordinated combination of reward system and prefrontal cortical structures occurs. Controlled attention, deliberate decision, and execution of prosocial actions can come about in this way. Controlled mentalization, however, fails in states of high or extreme arousal. Here, the predominantly subcortically mediated reaction pattern of “*fight-flight*” and danger-oriented vigilance prevails. This is essentially mediated by D_2_-dopaminergic, α_1_-adrenergic and serotonergic neurotransmission, whereas prefrontal-cortical systems are severely restricted in their functionality (50, 51).

In individuals with an insecure attachment pattern the threshold of a switch from a controlled to an automated mode of mentalization is usually significantly low. It is an apparent paradox of human development that precisely insecurely attached people, who habitually show possible partners a heightened distrust and great ambivalence, strongly activate their unconscious attachment system in situations of strong psychological or social stress. This is especially true for people with “*anxious-ambivalent*” attachment. On a neurobiological level, a strongly activated attachment system goes along at the same time with a deactivation of two other neural systems, which would be of great service to mature mentalization. On the one hand, it is a network consisting of the medial parts of prefrontal cortex, inferior parietal cortex, medial temporal cortex and posterior cingulate cortex. Under normal circumstances, this neural system organizes focused attention, episodic, especially autobiographical long-term memory and, in the case of positive and negative affect states, a special, emotion and cognition-integrating function. On the other hand, it involves a network that functionally connects the poles of the temporal lobe, temporoparietal connection, amygdala and medial prefrontal cortex, conveying judgments about social trustworthiness, moral judgments, a theory of mind and attentiveness to one’s own feelings (39).

The attachment system activated in insecurely attached individuals therefore functions somewhat automatically. Although this automatic mode allows for rapidly available mentalization steps, these are usually undifferentiated and global. The automatic mode aims to induce and share intense emotions in the current relationship situation. In cognitive orientation and affective evaluation, it primarily relies on external features such as the currently shown emotional facial expression of a partner, without performing a differentiated intrapsychic motivational analysis. It is therefore easy to jump to hasty conclusions about the significance of a current situation. In an automatic reaction mode a representation system shared by self and object is predominantly active. In this case, it may become increasingly difficult to correctly recognize and distinguish whether the affects belong to the self or to the other. A major emphasis of the externally perceived affect expressions of the other easily results in an uncontrolled affect transferal. It is important to notice that with the overactivated attachment system, the “*fight-flight*” system is also upregulated and abrupt changes may occur between panicked timidity and aggressive hostility (39)**.**

Individuals with “*anxious-avoidant*” attachment patterns have learned to habitually classify social contacts as potentially dangerous and unsettling and thus prefer to avoid them. Instead, they may have developed compensatory techniques to strengthen their autonomy, independence and self-sufficiency. In states of psychological stress, they activate their implicit attachment system to a much lesser extent. They succeed in maintaining sufficient cognitive control in the respective situation for a longer period. However, their retrievable cognitive self and object schemata are usually rigid, with a strong bias and only poorly suited for constructive conflict resolutions in delicate, interpersonal relationships. It is also obvious that these strategies of deactivating the attachment system at the same time require enormous defensive energy. The associated increased intra-organism stress level may contribute to significant mental and physical health risks in the long term (39).

### Attachment Trauma

According to Allen, (7), attachment trauma translates to the overwhelming experience of feeling alone in the midst of an unbearable emotional state or, worse, realizing that the attachment person itself is the cause of overwhelming distress. Exposition to a traumatizing attachment figure impairs the basic ability to achieve a secure attachment at all. It leads to the formative expectation that all relationships are dominated by mistrust. Fonagy et al. (40) refer to a complex situation: attachment trauma very often is cumulative, not infrequently persistent. It causes a shattering emotional distress and undermines the ability to effectively regulate this emotional distress. And it is usually incompatible with the development of a mature mentalization. Attachment trauma may occur in the form of a basic interpersonal neglect (*omission trauma*) or in the form of physical, mental or sexual abuse (*commission trauma*). In many cases, both trauma types are combined. Attachment trauma often leads to a “*disoriented- disorganized*” attachment. A disorganized attachment pattern in turn imparts an increased risk of further abuse and neglect. Attachment traumata, however, do not happen in an empty social context. Massive problems in parental care are empirically associated with numerous unfavorable psychosocial stressors, e.g. severe chronic marital conflict, perinatal loss of a previous baby, handicapped baby, postpartum depression/psychosis, parental psychiatric morbidity and violent environment (52, 53).

### Key Theme in Attachment Trauma

Attachment trauma forces the child into a developmental dilemma with no way out, a constant “*horror without resolution*” (54): Traumatic anxiety, fear, or panic is associated with the presence of a central attachment figure. However, this situation inevitably activates the natural “attachment system” and provides a motivation to find presumed safety in the person through an intense search for closeness, which may further increase emotional distress. This indissoluble developmental paradox consists in maximum activation of an approaching tendency to the traumatizing attachment figure with simultaneous activation of the escape system without, however, being able to achieve consistent behavioral management. One could generally conclude that attachment traumata not only mediate damaging effects due to the specific traumatic impact, they cause even more profound psychological wounds by incompatibly colliding with the acquisition of the ability of a trusting relationship in itself and with the chance for unrestricted and autonomous self-development (7, 55).

These antagonistic behavioral desires that continually determine a child’s disoriented-disorganized attachment may become apparent in the developmental psychological observation paradigm of “*separation and reunification*.” The complete lack of a behavioral plan on the part of the child to consistently and effectively deal with the typical emotional challenges in this experimental situation may correspond to an uncontrollable alternation between hostile intrusiveness and helpless withdrawal in the specific interaction on the part of the mother, when traumatic experiences are primarily associated with her (56). Again, in other social interactions with the attachment figure, the child themselves may actively replicate the incompatible parental care behavior in a desperate bid to regain emotional control of the actual relational situation. The child struggles to resolve its dilemma of closeness and distance in dealing with the attachment person by alternating between a controlling-punishing versus controlling-caring behavioral pattern (57).

### Disoriented-Disorganized Attachment Pattern and Increased Risk of Further Traumatization

Established insecure attachment patterns are empirically associated with a higher rate of traumatic events and subsequent sequelae of trauma (58, 59). For disoriented-disorganized attachment patterns, this increased vulnerability to traumatization applies above all to other attachment contexts. They aggravate the associated developmental deficits of mentalization, as outlined above, for insecure attachment patterns (40, 60):

Further trauma has a disastrous impact on affective and socio-cognitive development. Sexual or aggressive exposures of abuse by a parent, for example, are particularly devastating if they are based on a previous relational context of emotional neglect. Traumatic experiences often fatefully stabilize existing identity diffusion by structuring the emotional life *via* splitting or dissociation. They may promote “*identification with the aggressor*” and, as a result, may create intrapsychic relational representations of “*perpetrators and victims*” in rapid reversals. This does not just mean an increased risk of re-traumatization. It also encourages a reverse tendency towards outward victimization. However, this dominant behavioral pattern is based on a massive obstruction of general mentalization functions. Due to the overwhelming destructive affects in the trauma itself, it is often not possible to correctly record the event between perpetrator and victim in the sense of an identifiable object-subject relation that can be represented in this way. Rather, the trauma is encoded as the destructive affect state of an “*adualistic monad*” (61). The result may be a malignant introjection that constantly presses for externalization in concrete relationships. As a result of trauma-related dissociation, it can thus neither be self-reflexively assessed nor independently modified. Although hypervigilance towards the emotional facial expressions of potential perpetrators almost predominates, there is also a fundamental inhibition or refusal to empathize with or even to recognize the mental state of perpetrators at all. In a dissociative altered state of consciousness, the various aspects of a risky situation can often not be noticed. Thus, it often happens that object-related external perception and blocked self-reflexive inner attitude may express a distancing towards a potential offender, while on an unconscious or dissociated physical signal level, an attachment-inherent search for proximity may be effective. This contradictory behavior reinforces the already established, disoriented-disorganized attachment pattern and maintains a strong risk of further traumatization.

### Disoriented-Disorganized Attachment Pattern and Trauma-Induced Dissociation

Intensive clinical and neuroscientific research has led to the following insight into some of the more debilitating consequences of attachment trauma: Posttraumatic processing not only follows the known psychological and neurobiological pathways in the transition to post-traumatic stress disorder (PTSD), mediating the typical symptom clusters of trauma-related intrusive recall, avoidance and autonomic hyperarousal as a result. It is also essentially determined by trauma-induced dissociative processes (62, 63). Dissociative symptoms result, on the one hand, from a failure to integrate trauma-related information (“*compartmentalization*”), and, on the other hand, from an increased use of the evolutionarily anchored protective mechanism of depersonalization and derealization (“*detachment*”) (7, 55). On a perceptive and cognitive level, they affect key aspects of the psychological and psychosocial identity and the post-traumatic self. They also contribute to fragmentation in object perception. These trauma-induced dissociative processes also directly affect the systems of mentalization and empathy. They lead to deficits at an even greater extent, as they have been described in the context of uncertain bond patterns above.

About one third of all PTSD patients, especially those with a history of early attachment trauma, present a special dissociative type. Nevertheless, these patients do not permanently live within traumatically-dissociative altered states of consciousness, but commute depending on the intensity and frequency of traumatization and current situational pressures between the poles of a prominently dissociative experience and a normal, waking consciousness. In the four phenomenological areas of time experience, intentionality of mental processes, body awareness and emotional regulation, prominent post-traumatic dissociative psychopathologies may be described in a differentiated manner (64, 65):

1. In the *time dimension* of our consciousness, it is possible for us to voluntarily turn our eyes from the moment of the present into the past as well as into the future. Here, the autonoetic knowledge, which is bound to an intact functionality of the autobiographical memory, can clearly differentiate between a current experience, a retrospective memory, or a future-oriented presentation. In traumatically altered states of consciousness, this confident performance of the self may be completely suspended by flashbacks and fixed to an involuntarily revived traumatic timeline. Time experience can be fundamentally changed in this situation. It is stretched almost timelessly in some cases, but also often perceived as accelerating disquietingly on the other hand. Even in normal, i.e. not dissociative, altered states of consciousness of everyday life, intrusive recollections may occur and cause great emotional distress. These traumatic recollections, however, do not necessarily alter the autonomic consciousness of the present, the past and the future, although they may influence it significantly.
2. Our consciousness is *intentionally* created in relation to the environment and is usually organized in distinct subject-object relations. This first-person perspective can be lost in traumatically dissociative altered states of consciousness, when one’s own thoughts or memories can only be perceived in the form of voices. Here, the self-referential point of view of the conscious experience is qualitatively changed into a second person perspective. In the normal waking consciousness, numerous negative self- and object-referential cognitions, as well as evaluations related to trauma, may be present as well. Even if a person’s basic self and object schemata are shaken to the very core of security, trust, self-worth, dependency, autonomy, control, intimacy, causality, and hope, the basic structure of personal identity, however, is usually not split in this state.
3. In the dimension of *body awareness*, states of depersonalization on the one hand and autonomous hyperarousal on the other can appear on both poles. Depersonalization in its pronounced, traumatic-dissociative form very often involves states of separation of the externally perceptible, externally perceived own body in a third person perspective and a self that is separated from bodily sensations, only mentally observing oneself (“*out-of- body experiences*”). This experience also indicates a fundamental modification of the identity structure. In conditions of autonomic hyperarousal triggered by normal waking consciousness, agonizing and disturbing body sensations in turn can completely control acute life and may be associated with the fear of loss of control. Nevertheless, there is no doubt that it is the subject’s own body which is currently in turmoil. An intermediate position is occupied by those cases of dissociative disorders of the motor or the sensory system, in which the action or the sensibility in parts of the body or the body representation is withdrawn from a deliberate control of the self.
4. Finally, in the dimension of *emotional regulation*, two poles are determined in an analogous manner by a state of total emotional numbness on the one hand and by conditions of trauma-related affective states of overwhelming anxiety, horror, panic, shame, and guilt, on the other.

These four phenomenological dimensions of traumatic-dissociative psychopathologies are also closely associated with significant deficits of empathy and mentalization.

At the neurobiological level, there is currently no clear picture regarding a disoriented- disorganized pattern of attachment, as seems to be possible with secure and insecure attachment patterns. Disoriented-disorganized attachment patterns are primarily conceptualized in the context of diverse attachment traumata. Neurobiological research approaches have so far been performed mostly in adults who had severe trauma either in early developmental stages or later on in life, often in adolescence or adulthood; they exhibited a series of mental disorders that were to be conceptualized as associated clinical sequelae, such as a PTSD, complex PTSD, dissociative disorders, serious personality disorders, in particular of the borderline-type, but also variants of chronic depression, anxiety, somatization syndrome, chronic suicidal behavior or substance-related disorders. Significant psychopathological, psychodynamic and trauma-related overlaps are to be noted between these different clinical states (66). In neurobiological investigations, a similar transdiagnostic view is gradually embraced. Findings previously associated with individual diagnostic categories, e.g. in neuroimaging, are now increasingly evaluated as a more general characteristic imprint of just these early trauma exposures (67).

Controlled neuroscientific research on toddlers with serious emotional neglect or various forms of emotional, physical or sexual abuse, however, defies medically-ethical standards and is mostly difficult to realize for practical reasons. Suitable animal models, which approximately simulate traumatization of the early attachment process, may present themselves as insightful approaches to bridge some gaps in the understanding of the effects of early traumatization. Some aspects can be summarized as follows (68):

- Effects of early trauma involve fundamental changes at all levels of analysis, ranging from cellular signaling to behavioral expression. They include a variety of neurotransmitter systems, mechanisms of stress mediation (e.g., HPA axis, neuroinflammation), and numerous neural brain circuits. Various affected brain regions have their own maturation and development pathways and, in turn, are responsible for a myriad of distinct behaviors with their own independent developmental trajectories. Some brain areas encode traumatic information, which may later lead to behavioral problems. Of major importance in this context is the fact that some brain regions play a key role in the mediation of traumatic experiences in adulthood: For example, the amygdala, hippocampus, and especially the prefrontal cortex are still highly immature in early stages of development. However, early traumatization may pave the way for the further development of these structures, but their effects will only become apparent much later as atypical stress reactions. Traumata in these early stages of development primarily affect the neural attachment system.
- The neural attachment system is evolutionarily designed to inherently force a baby to maintain stable contact with an attachment figure for survival reasons, even under traumatic circumstances. Even the most adverse, painful experiences may be integrated into this primary relationship form. From birth, a rich noradrenergic neurotransmission (locus coeruleus) is available for these basic learning processes. Structural and functional immaturity of those brain regions organizing avoidance behaviors in later stages of development, such as the amygdala, forestall an efficient behavioral strategy of being able to withdraw from a traumatizing attachment figure at this early stage. On the contrary, the relationship with the dominant attachment figure may be particularly robust despite the low quality of parental care and repeated traumatic exposures.
- Nonetheless, those systems that are still largely immature in early stages of development have a major impact on later stages of maturation and development. They are prominently demasked at times when there are additional stressors or traumata. With an overactive system of threat perception and evaluation (cortico-amygdalar), a significantly reduced reward system (cortico-basal ganglia) and a severely restricted higher-cortical control and executive system (prefrontal cortex), there may be transposed not only massive vulnerabilities from the early traumatic developmental history into later stages of life, as regards further stressors and traumatas, but also drastically reduced chances of successful processing. Current empirical data of neuroimaging emphasizes the main modes of pathological processing of traumatic experiences, the mode of “*autonomous hyperarousal*” on the one hand and “*dissociative depersonalization and derealization*” on the other (see above):

1. In provocation paradigms aimed at recalling traumatic experiences, the most consistent neural pattern in *autonomous hyperarousal mode* is a hyperactive amygdala, hyporeactive ventral prefrontal cortex, hyperactive dorsal anterior cingulate (dACC), and hyperactive insular region. In the general, clinical approach of understanding pathological forms of post-traumatic processing, these findings illustrate that in individuals with PTSD a shift in dominant central nervous regulation from prefrontal structures to amygdala-centered control occurs (69).
2. In a prevailing view of *dissociative mode of depersonalization* during confrontation with traumatic memories, the findings can be summarized to the following neuronal activation network: Compared to a control group, a stronger activation is found in the upper and middle temporal gyri, parietal and occipital lobes, middle frontal gyrus, medial prefrontal cortex and ACC, with overall reduced activity of the amygdala. The affective, cognitive, and body-related variants of dissociative alienation mediated thereby acutely succeed in containing autonomic-nervous hyperactivity in the context of a traumatic memory. However, this creates major problems in the effective processing of traumatic experiences from a long-term perspective (64).

### Some Critical Comments

Some critical remarks on the described psychobiological contexts of attachment, socio-affective and cognitive development should be made. The theoretical models of affective and cognitive empathy and mentalization, as developed especially in the working group around Peter Fonagy, have enormously enriched the clinical handling of patients with severe mental disorders with insecure attachment patterns and impaired functions of their empathy and mentalization capacity. They have also contributed significantly to the empirical clarification of psychological and interpersonal mechanisms of affect regulation and mentalization within a developmental context (70, 71). The attachment patterns acquired in the early childhood interactions of the mother-child dyad have a significant and probably also a lasting significance in the mediation, maintenance or endangerment of psychosomatic health in later stages of life. But relations are neither unilinear nor monocausal. It seems to be well established empirically that a predominantly successful interactive coordination between mother and baby encourages the establishment of a secure attachment pattern in the child and also promotes its development to more mature levels of cognitive-affective mentalization (40). It also seems to be empirically validated that a secure attachment pattern of the mother is a strong predictor for the quality of the child’s attachment pattern, just not unilinear and monocausal. A well-bound mother may also fail due to the multiple challenges in her relationship with her baby, her baby’s primarily difficult temperament, or other adverse family and social circumstances, traumas, existential disasters etc. the fundamental voting processes continue to disturb. In the former case, it can be seen that bidirectional influences are present in early attachment behavior, that is, the baby itself, e.g. exercises a potentially pathogenic effect on the behavior of the mother through a constitutional handicap and thereby reduces the quality of achievable attachment security. In the second case it is emphasized that an exclusive focus on the mother-child dyad greatly simplifies the multiple influencing factors of the family and social environment. Empirical studies, theoretically based on transactional development models and statistically based on complex analytical techniques, strongly demand this viewpoint (8).

The central theoretical assumption, that distinct bonding patterns from early mother-child interactions once formed are fundamental, lifelong blueprints for shaping future relationships, must be differentiated. A broader psychosocial and cultural context is indicated. Important transformations in later stages of development, especially in adolescence, cannot be explained solely by the dynamics of early mother-child interactions. Rather, numerous biopsychosocial factors influence transformations during this particular developmental period (72–74). In this context, the empirical finding should also briefly mention that some adults in the commonly used Adult Attachment Interview achieve a secure attachment status, but report significant problems from their early relationships with parents. In contrast to people who receive both the current classification of a secure bond and affectionate contacts with their parents (“continuous secure”), this attachment status is referred to here as “earned secure.” It seems to indicate a developmental transition from initial “insecure” to “secure” later (75). The relations are again complex. They are definitely not to be clarified in retrospective cross-sectional studies, but at best in prospective longitudinal studies (76). Confounding variables such as current depressive or anxious symptoms with a memory bias has to be considered (75), compensating social contacts with important other persons besides the parent figures (77, 78), influences from adolescent development dynamics with a critical reflection on the early relational history with the parents (79), a favorable current relationship status in adult life with positive corrective emotional experiences (80) and important modifying effects in interim psychotherapy (81) is notable.

Psychobiological characteristics in the context of early acquired attachment status may be considered as significant protective and resilience-promoting factors in mental health and somatic health, even in adult life. Similarly, those psychobiological characteristics acquired in an insecure or disorganized attachment context are likely to be life-long effective trait variables with significant negative consequences for increased mental and somatic disease risks (82, 83). However, the connections are again to be conceptualized as highly complex (84). This should also be considered for the neurobiological findings presented above, which have been grouped in the theoretically underlying framework of “safe” versus “insecure” and “disorganized” attachment types with respect to impaired mental functions of empathy and mentalization (39). The majority of these studies have been performed on adult patient samples with different psychopathological formations and correlated unsafe/disorganized binding types. However, the neurobiological findings, often obtained with methodologically very different examination paradigms, leave open the question of, for example, early traumatic events per se, a diagnostic status of post-traumatic sequelae acquired at an earlier or later stage of development, or the current psychopathological status with potentially multiple co-morbid mental disorders. Disorders and personality disorders have decisively determined the neuronal activation patterns found. The assignment to distinct types of binding must not take place in a unilinear or monocausal sense. Nonetheless, the findings give a significant insight into the neurobiological mediation mechanisms of impaired mental functions of empathy and mentalization of patients who are currently under massive stress and presently have different attachment patterns.

## Conclusion

The most fundamental characteristics of the conditio humana are attachment and the ability to form stable bonds with significant others. An evolutionary principle deeply rooted in phylogeny underpins this drive. John Bowlby’s research can only be regarded valid if the attachment system is regarded as a primary motivational system in the individual development of human beings. Initially, this principle unfolds in the interactions of the early mother-child dyad. In its evolutionary-biological composite parts, it usually guarantees a particularly intensive emotional exchange between the two partners, which should convey safety, security, value and trust and finally effective self-regulation. The typical psychosocial experiences here not only shape the neurobiological organization and structure of the further differentiating brain of the child, the particular quality of these early relationship experiences is also crucial for the further affective, cognitive and social development in the context of his/her brain development. An acquired attachment pattern is intimately linked to the capacity for empathy and mentalization of the growing child, both psychological skills that will determine his/her future relationships. While secure attachment provides a vital foundation for healthy development, an insecure and, above all, a disoriented and disorganized attachment is associated with increased risks for numerous mental and somatic diseases. Although traumata in the early attachment period provide a serious legacy, both on psychological and interpersonal as well as neurobiological levels, for further life and personal development opportunities, this is not an absolutely irreversible fate for one’s own existence and subsequent generations, as impressively shown by special psychotherapeutic approaches (81).

## Author Contributions

TL, HU, and H-PK wrote the manuscript. All authors gave their consent for publication.

## Conflict of Interest

The authors declare that the research was conducted in the absence of any commercial or financial relationships that could be construed as a potential conflict of interest.

The handling editor is currently co-organizing a Research Topic with one of the authors HU, and confirms the absence of any other collaboration.
